# Adaptation of *Pseudomonas aeruginosa* in Cystic Fibrosis Airways Influences Virulence of *Staphylococcus aureus In Vitro* and Murine Models of Co-Infection

**DOI:** 10.1371/journal.pone.0089614

**Published:** 2014-03-06

**Authors:** Rossella Baldan, Cristina Cigana, Francesca Testa, Irene Bianconi, Maura De Simone, Danilo Pellin, Clelia Di Serio, Alessandra Bragonzi, Daniela M. Cirillo

**Affiliations:** 1 Emerging Bacterial Pathogens Unit, San Raffaele Scientific Institute, Milan, Italy; 2 Infection and Cystic Fibrosis Unit, San Raffaele Scientific Institute, Milan, Italy; 3 University Centre for Statistics in the Biomedical Sciences, Università Vita-Salute San Raffaele, Milan, Italy; The Hospital for Sick Children and The University of Toronto, Canada

## Abstract

Cystic fibrosis (CF) airways disease represents an example of polymicrobial infection whereby different bacterial species can interact and influence each other. In CF patients *Staphylococcus aureus* is often the initial pathogen colonizing the lungs during childhood, while *Pseudomonas aeruginosa* is the predominant pathogen isolated in adolescents and adults. During chronic infection, *P. aeruginosa* undergoes adaptation to cope with antimicrobial therapy, host response and co-infecting pathogens. However, *S. aureus* and *P. aeruginosa* often co-exist in the same niche influencing the CF pathogenesis. The goal of this study was to investigate the reciprocal interaction of *P. aeruginosa* and *S. aureus* and understand the influence of *P. aeruginosa* adaptation to the CF lung in order to gain important insight on the interplay occurring between the two main pathogens of CF airways, which is still largely unknown. *P. aeruginosa* reference strains and eight lineages of clinical strains, including early and late clonal isolates from different patients with CF, were tested for growth inhibition of *S. aureus*. Next, *P. aeruginosa/S. aureus* competition was investigated in planktonic co-culture, biofilm, and mouse pneumonia model. *P. aeruginosa* reference and early strains, isolated at the onset of chronic infection, outcompeted *S. aureus in vitro* and *in vivo* models of co-infection. On the contrary, our results indicated a reduced capacity to outcompete *S. aureus* of *P. aeruginosa* patho-adaptive strains, isolated after several years of chronic infection and carrying several phenotypic changes temporally associated with CF lung adaptation. Our findings provide relevant information with respect to interspecies interaction and disease progression in CF.

## Introduction

Chronic airway infections and inflammation cause progressive lung disease and are the leading causes of mortality in patients with cystic fibrosis (CF) [1]. CF disease is characterized by the accumulation of secretion in the lungs and by a decreased mucociliary clearance that lead to an impaired ability to defeat bacterial infections. The viscous CF lung secretions provide an environment that protects bacteria from the assault of antibiotics and immune cells, thus favoring colonization and persistence. CF patients have a unique set of bacterial pathogens that are frequently acquired in an age dependent sequence [2]. The most frequently cultured organisms from the respiratory tract of young children are *Staphylococcus aureus* and non-typeable *Haemophilus influenzae*. Later, as the patient ages, infection progresses to involve opportunistic pathogens such as *Pseudomonas aeruginosa* and *Burkholderia cepacia*.

It is now becoming clear that the different bacteria coexisting in CF airways have a mutual interaction and contribute to the pathogenesis of the disease [3], [4]. In a context that involves a complex polymicrobial community a single-species microbial analysis could be inadequate, as different microbes within the community can interact each other and the resulting infection pathogenesis differs from that in infections caused by the component species individually [3], [5]. Chronic bacterial infections associated with CF lung disease have been studied by a range of culture-independent profiling methodologies [6]–[12], and each approach has revealed greater microbial diversity than was previously recognized. Overall, the results of these studies suggest that the polymicrobial nature of CF infections could play a key role in driving disease and response to therapy and, in turn, significantly impact upon clinical outcomes [1], [7], [13]. Nevertheless, very little is known about the role of interspecies interactions in the pathogenesis of the CF lung disease [14], [15].

The Gram-positive bacterium *S. aureus* is the pathogen most commonly isolated in nasopharyngeal samples from young children with CF, and in the preantibiotic era, many CF patients succumbed to *S. aureus* infection [16]. Recent data demonstrate an increase in *S. aureus* infections in the CF population, not only in the US but also in Europe, with methicillin-resistant *S. aureus* (MRSA) strains being on the rise [17], [18], reflecting the overall increase in prevalence and epidemiologic changes in the general population [19], [20].

Of the multiple opportunistic bacteria that may infect CF patients, the Gram-negative bacterium *P. aeruginosa* is considered to be the most significant as it has clearly been linked to worsening of the pulmonary status [21]. Despite intensive antibiotic treatments, *P. aeruginosa* infections are difficult to eradicate [22]. The antibiotic treatment may favor the emergence of antimicrobial drug resistance. One of the most striking characteristics of *P. aeruginosa* chronic lung infection in CF patients is indeed the co-existence of multiple phenotypes that are highly resistant to any chemotherapy treatment [23].

Although *S. aureus* colonization/infection usually precedes chronic colonization of the respiratory tract by *P. aeruginosa*, it continues into adulthood, when 51% of patients become culture positive for *S. aureus* [24]. Both organisms are commonly co-isolated from CF respiratory cultures and it has been shown that risk factors for initial *P. aeruginosa* airway infection in patients with CF include *S. aureus* pre-colonization [25], [26]. In addition, both species are able to shift between a planktonic (free-living) life style to surface-attached communities known as biofilms during chronic infections. In human diseases including CF, biofilm-related infections are directly correlated with dramatic increases in antibiotic resistance [27], [28], [29].

In this study, we aimed to explore the interactions between *S. aureus* and *P. aeruginosa* by using *in vitro* and murine models of pneumonia. During chronic infection, *P. aeruginosa* undergoes numerous selective pressures ranging from antibiotic treatments, host immune response and interactions with other microorganisms leading to the development of patho-adaptive lineages. The adaptation of *P. aeruginosa* to the CF niche selects for clones with reduced virulence in multi-hosts models [23], [30]. We focused our attention on the reciprocal influence of *P. aeruginosa* and *S. aureus* and on understanding how *P. aeruginosa* adaptation to the CF lung may interfere with *S. aureus* interaction. Using a collection of longitudinal strains isolated from CF patients, we showed that *P. aeruginosa* strains out-competed *S. aureus*. This effect was associated with *P. aeruginosa* early strains, which in acute infection present higher virulence. On the contrary, *P. aeruginosa* late adapted strains showed reduced or abolished capacity to outcompete *S. aureus*. This work provides key results on lung pathogenicity caused by multi-bacterial infection.

## Results

### *P. aeruginosa* early and late clonal variants differently influence growth of *S. aureus*

Eight lineages of *P. aeruginosa* strains, including 12 early (early group) and 12 late (late group) clonal isolates from different patients with CF were tested for growth inhibition of *S. aureus* Newman and SH1000 strains on agar surfaces [25]. In particular, late *P. aeruginosa* strains selected for this study were collected over a period of 16.3 years and carried several patho-adaptive traits, including mucoid and hypermutable phenotypes (Table 1) as reported previously [23], [31]. In addition, PAO1 and PA14 *P. aeruginosa* reference strains, which show phenotypic traits characteristic of early isolates [23], were also included.

**Table 1:** *In vitro* growth inhibition of *S. aureus* and *P. aeruginosa*.

*P. aeruginosa* spot	*S. aureus* lawn (Newman) (inhibition halo)	*S. aureus* lawn (SH1000) (inhibition halo)
PAO1	strong (24.5 mm)	strong (20 mm)
PA14	strong (22 mm)	strong (19 mm)
SG1	strong (23.5 mm)	strong ( 22.5 mm)
SG57*	strong (23 mm)	strong (20.5 mm)
SG58*	weak (14.5 mm)	weak (15 mm)
NN2	weak (15 mm)	weak (14 mm)
NN83# *	no (9 mm)	no (9 mm)
BT1#	weak (15 mm)	weak (14.5 mm)
BT2	weak (15 mm)	weak (15 mm)
BT72*	no (9 mm)	no (9 mm)
BT73*	weak (13.5 mm)	weak (13 mm)
AA2	very strong (27 mm)	strong (20.5 mm)
AA43*	no (9 mm)	no (9 mm)
TR1	weak (13.5 mm)	weak (13.5 mm)
TR2	strong (20 mm)	strong (20 mm)
TR66*	weak (11.5 mm)	weak (11.5 mm)
TR67*	no (9 mm)	no (9 mm)
MF1	weak (15 mm)	strong (21 mm)
MF2#	strong (21 mm)	strong (18.5 mm)
MF51*	no (9 mm)	no (9 mm)
KK1	weak (12 mm)	no (9 mm)
KK2	very strong (27 mm)	very strong (26.5 mm)
KK71*	no (9 mm)	no (9 mm)
KK72*	no (9 mm)	no (9 mm)
BST2	weak (15 mm)	weak (14.5 mm)
BST44# *	weak (14.5 mm)	weak (12.5 mm)

Twenty-four *P. aeruginosa* isolates were collected from eight individuals with CF (SG, NN, BT, AA, TR, MF, KK, BST) at the onset of chronic colonization (numbered 1-2) or after 4.5–16.3 years of colonization (numbered 43-83). PAO1 and PA14 were included as reference strains. 5 µl spots of *P. aeruginosa* overnight cultures, normalized to 0.5 OD, were added to *S. aureus* lawn (normalized to 0.5 OD) on Mueller-Hinton agar and incubated overnight at 37°C. The table summarizes the results obtained: “weak inhibition” indicates an inhibition halo ≤15 mm; “strong inhibition” indicates an inhibition halo >15 mm and ≤25 mm; “very strong inhibition” indicates an inhibition halo >25 mm; “no inhibition“ indicates absence of inhibition halo (9 mm is the diameter of the *P. aeruginosa* spot).

* Indicates mucoid phenotype.

# Indicates hypermutable phenotype. For statistical analysis see “Results”.

As shown in Table 1, growth of *S. aureus* Newman and SH1000 strains was inhibited by PA14 and PAO1 *P. aeruginosa* reference strains and by 100% (12/12) and 91.6% (11/12) of *P. aeruginosa* early strains respectively in co-culture. The only exception was the strain KK1 which was previously described as different in terms of virulence potential from KK2, isolated at the same time point [23]. The strength of inhibition of *S. aureus* in some cases differed within clonal lineages (TR1 vs TR2; MF1 vs MF2; KK1 vs KK2).

Differently from *P. aeruginosa* early strains, 58.4% (7/12) of the late strains had no effect on growth of *S. aureus* Newman and SH1000 strains. These *P. aeruginosa* strains belonged to six different lineages (NN, BT, AA, TR, MF, KK) indicating presence of at least one *P. aeruginosa* strain unable to inhibit *S. aureus* growth in the majority of CF patients (75%: 6/8). The other two *P. aeruginosa* lineages (SG and BST) (25%: 2/8) inhibited *S. aureus* growth although to a lesser extent when compared to early strains. Late *P. aeruginosa* strains within the same lineage also differed with regard to the strength of *S. aureus* growth inhibition (SG57 vs SG58; BT72 vs BT73; TR66 vs TR67), indicating a diversification of the bacterial population during chronic infection as demonstrated for other virulence traits [23], [32]. The average inhibition halo of late group was 11.6 mm versus Newman and 11.3 mm versus SH1000, while the average inhibition halo of early group was 18.3 mm versus Newman and 17.4 mm versus SH1000. Late group showed a statistically significant effect in reducing levels of inhibition (regression parameter = −6.76 versus Newman and regression parameter = −6.24 versus SH1000) with p<0.01 for both settings. This data indicated that, as a group, late *P. aeruginosa* strains differ significantly from early strains in their capacity to inhibit *S. aureus* growth, suggesting a trend of *P. aeruginosa* patho-adaptive variants to influence the growth of *S. aureus*.

On the contrary *S. aureus* did not exert any effect on the growth of *P. aeruginosa* (Table S1).

### Competition between *S. aureus* and *P. aeruginosa* in planktonic co-cultures

**Figure 1:**
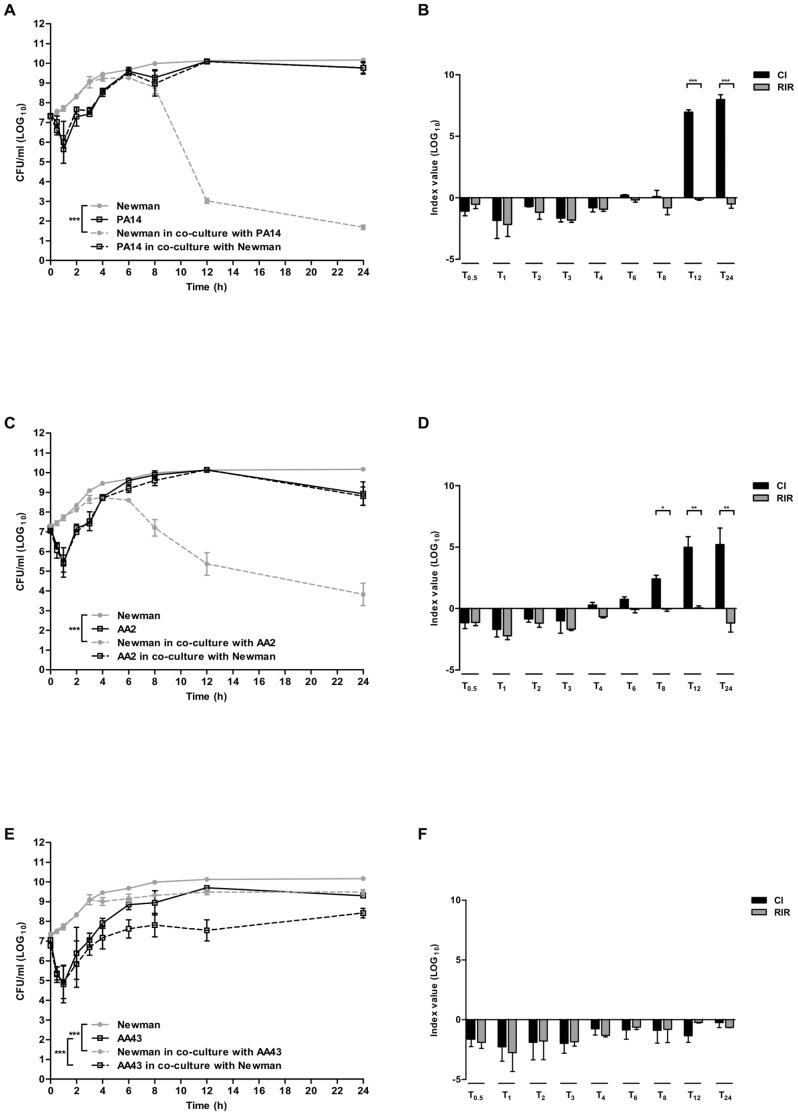
Single and dual species batch growth curves and competition index values. *S. aureus* strain (Newman) and *P. aeruginosa* strains (PA14 and two clinical early and late isolates from a CF patient AA2 and AA43) were grown for 24 hours in BHI in single culture and in co-culture after inoculation at equal ratio from mid-exponential phase pure cultures. Growth rate was monitored by colony count after plating on selective media for both species. Results are represented as the mean of values obtained from three independent experiments. The error bars indicate the standard deviations. A nonlinear mixed-effect model was fitted, using a four-parameters logistic regression function. Panel A: growth curves of Newman in pure culture and in co-culture with PA14; Panel B: Competition index (CI) and Relative Increase Ratio (RIR) calculated from single and dual cultures of Newman and PA14; Panel C: growth curves of Newman in pure culture and in co-culture with AA2; Panel D: CI and RIR calculated from single and dual cultures of Newman and AA2; Panel E: growth curves of Newman in pure culture and in co-culture with AA43; Panel F: CI and RIR calculated from single and dual cultures of Newman and AA43. Each value represents the mean of CI and RIR values from three independent experiments and the bars indicate standard deviation. Statistically significant differences in Student's t test and in nonlinear mixed-effect model are indicated by symbols when present: *: p<0.05; **: p<0.01; ***: p<0.001.

Next, we investigated the interactions between *S. aureus* and *P. aeruginosa* in planktonic growth by comparing the growth kinetics of the two organisms in co-culture to those obtained in pure culture. One reference *P. aeruginosa* strain PA14 and a pair of sequential strains from patient AA were selected. Figure 1A shows the growth curves of the reference *S. aureus* Newman and *P. aeruginosa* PA14 strains in single and dual cultures. PA14 maintained the same growth rate in pure culture and in co-culture, and had a significant negative effect on the overall trend of the growth of Newman (p<0.001). In order to have a clear comprehension of the differences in growth between *S. aureus* and *P. aeruginosa*, we calculated the Competition Index (CI), that allows to compare the differences in growth curve of mixed cultures, and the CI-like index, the Relative Increase Ratio (RIR), that compares the growth curves of the two species in pure culture (see Materials and Methods). As shown in Figure 1B, the CI of PA14 versus Newman was significantly different from the RIR in late exponential phase (12 h, p<0.001) and stationary phase (24 h, p<0.001) of growth, suggesting an inhibitory effect of *P. aeruginosa* on *S. aureus*.

Next, we explored the effect of *P. aeruginosa* strains isolated at the onset of chronic colonization (early strains) or several years after acquisition (late strains) from CF patients on growth of *S. aureus*. A pair of well characterized *P. aeruginosa* clonal strains isolated from CF patient were selected: the AA2 early strain and AA43 late adapted strain carrying several phenotypic changes in virulence factor production, and patho-adaptive mutations within the genome temporally associated with CF lung infection [23], [30], [33]. The growth of Newman was significantly inhibited by the presence of the early AA2 strain (p<0.001), while AA2 strain was not affected from the presence of *S. aureus* (Figure 1C). The CI of AA2 versus Newman was significantly higher than the RIR in late exponential phase (8 h, p<0.05 and 12 h, p<0.01, Figure 1D) and in stationary phase (24 h, p<0.01). On the other hand, Newman and the late strain AA43 interfered each other in co-culture, slightly but significantly reducing their growth rate compared to pure culture (p<0.001, Figure 1E). It is worth noting that AA43 inhibited the growth of Newman to a lower extent compared to AA2: while AA2 determined a reduction of 3, 5 and 6 log at 8, 12 and 24 h respectively, AA43 determined a reduction of less than 1 log at the same time points (Figure 1C and E). Being the competition reciprocal between the two species and considering their different growth rate in pure culture, the CI did not differ from the RIR (Figure 1F). Similar results were obtained using the same isolates of *P. aeruginosa*, AA2 and AA43, in co-culture with the reference *S. aureus* SH1000 (Figure S1) strengthening the results obtained with Newman. Taken together these data indicate that *P. aeruginosa* strains, including reference or those isolated at the early stage of chronic infection, can outcompete *S. aureus* in planktonic cultures. On the other hand, *P. aeruginosa* patho-adaptive strains lose this capacity over time.

### *S. aureus* and *P. aeruginosa* interaction in biofilm

**Figure 2:**
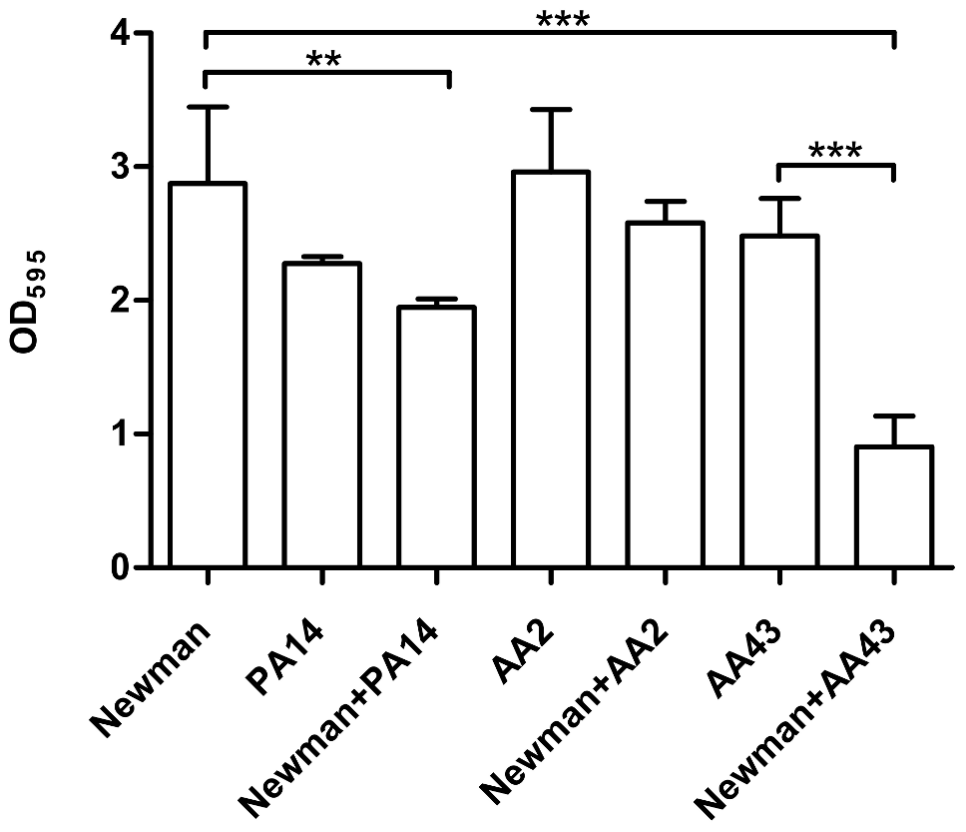
Biofilm formation by *S. aureus* and *P. aeruginosa* strains in single and dual cultures. Bacteria were grown overnight in 96-well flat-bottom microtiter plates in NB medium at 37°C either individually cultured or co-cultured at a 1∶1 ratio. Biofilm biomass was quantified by staining with crystal violet and absorbance measurements at OD 595 nm. The values represent the means of three independent experiments, and the bars indicate standard deviation. Statistically significant differences in Student's t test are indicated by symbols when present: **: p<0.01; ***: p<0.001.

In order to understand if the reciprocal interaction among the two species could affect their capacity to produce biofilm, we quantified the biofilm biomass of individually cultured or co-cultured at a ratio 1∶1 *S. aureus* and *P. aeruginosa* by staining with crystal violet. As shown in Figure 2, the results obtained from co-cultured pair of strains formed by Newman and PA14 revealed significantly lower level of biomass compared to Newman only (Newman vs Newman+PA14 p<0.01), but similar to that corresponding to PA14 alone. This data suggests an inhibitory effect exerted by *P. aeruginosa* on *S. aureus* biofilm formation. For clinical *P. aeruginosa* strains, while the OD value detected in the mixed biofilm formed by Newman and AA2 was not significantly different from both Newman and AA2 individually cultured, the OD value associated to the mixed biofilm formed by Newman and AA43 revealed significantly lower levels of biomass compared to both Newman (Newman vs Newman+AA43 p<0.001) and AA43 (AA43 vs Newman+AA43 p<0.001) individually cultured. This finding suggests a reciprocal interference of the two species, confirming the results of batch co-culture experiments.

**Figure 3:**
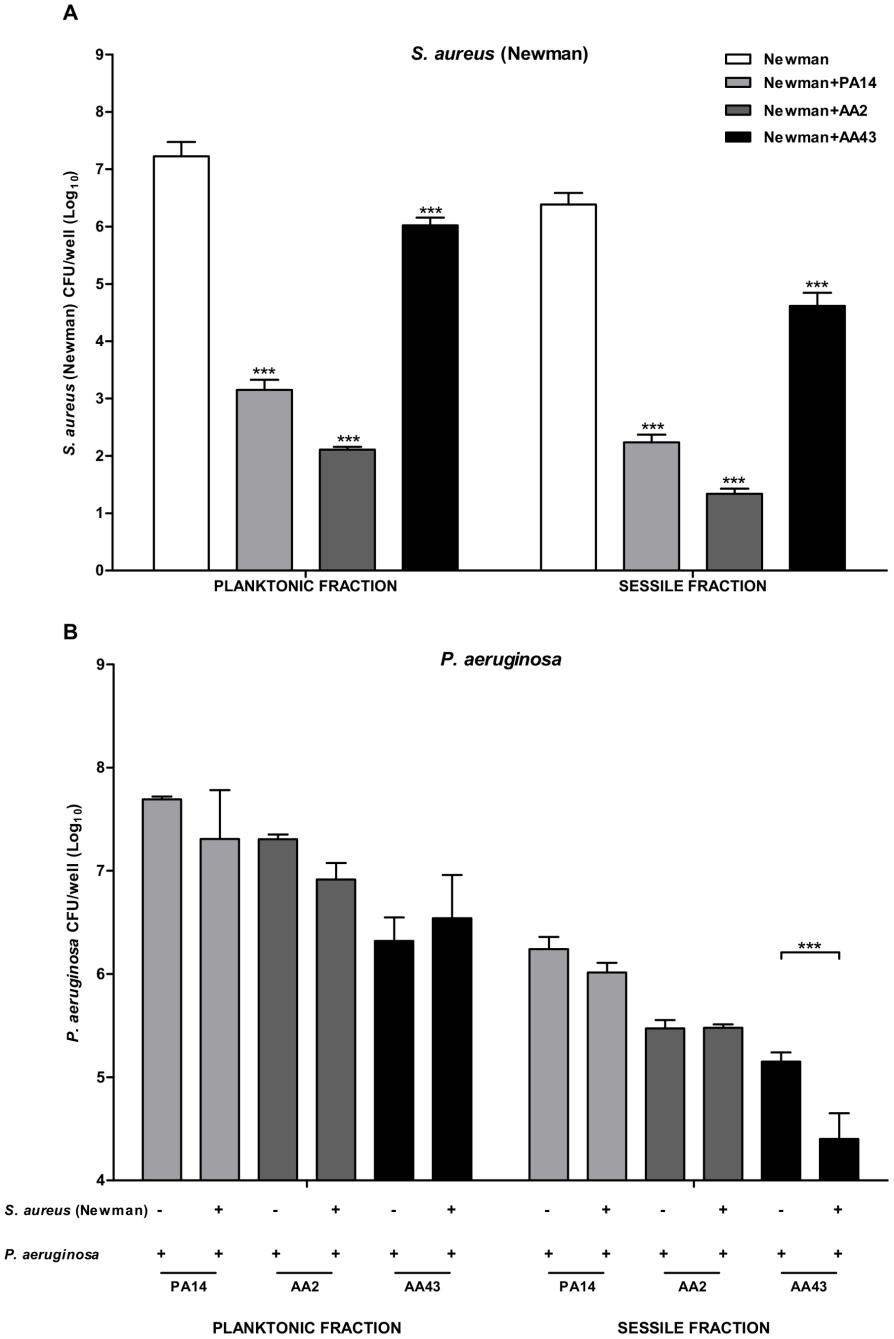
*S. aureus* and *P. aeruginosa* planktonic and sessile cells in single and dual cultures. Bacteria were grown overnight in 96-well flat-bottom microtiter plates in NB medium at 37°C either individually cultured or co-cultured at a 1∶1 ratio. CFU counts were determined in both planktonic and sessile fractions. Panel A: planktonic (left) and sessile (right) cells of *S. aureus* strain Newman in pure culture and in co-culture with *P. aeruginosa* strains PA14, AA2 and AA43. Statistically significant differences are referred to Newman in pure culture. Panel B: planktonic (left) and sessile (right) cells of *P. aeruginosa* strains PA14, AA2 and AA43 in pure culture and in co-culture with *S. aureus* strain Newman. The values represent the means of three independent experiments, and the bars indicate standard deviation. Statistically significant differences in non-parametric Mann–Whitney test are indicated by symbols when present: **: p<0.01; ***: p<0.001.

We also determined the amount of viable bacteria of each species in both planktonic and sessile fractions in single and dual cultures. In co-culture, we noticed that all strains of *P. aeruginosa* tested determined a reduction of the number of both sessile and planktonic Newman cells (p<0.001) (Figure 3A). In particular, the bacterial load of Newman in sessile fraction, when co-cultured with clinical early *P. aeruginosa* AA2, decreased of five log compared to pure culture, while the clonal late strain AA43 caused a lower (two log) reduction. A similar effect was observed also in planktonic fraction (Figure 3A) in agreement with batch co-culture data.

On the contrary, the presence of Newman had no effect on PA14 and AA2 growth in both planktonic and biofilm fractions, while it moderately inhibited the attachment to polystyrene and biofilm formation of the late *P. aeruginosa* strain AA43, confirming a reciprocal interaction between Newman and AA43 (Figure 3B, p<0.001).

**Figure 4:**
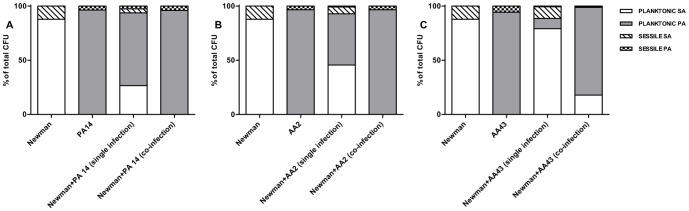
Percentage of planktonic and sessile cells in single and dual cultures. Bacteria were grown overnight in 96-well flat-bottom microtiter plates in NB medium at 37°C either individually cultured or co-cultured at a 1∶1 ratio. CFU counts were determined in both planktonic and sessile fractions and the percentage of S. aureus and P. aeruginosa in the two fractions of single and dual cultures was calculated. Panel A: percentages of planktonic and sessile cells of Newman in single culture (first histogram), PA14 in single culture (second histogram), Newman and PA14 in ideal co-culture if the 2 species would not interfere each other (third histogram, percentages have been calculated considering the values of the first and second histograms), and Newman and PA14 in co-culture (fourth histogram). Panel B: percentages of planktonic and sessile cells of Newman in single culture (first histogram), AA2 in single culture (second histogram), Newman and AA2 in ideal co-culture if the 2 species would not interfere each other (third histogram, percentages have been calculated considering the values of the first and second histograms), and Newman and AA2 in co-culture (fourth histogram). Panel C: percentages of planktonic and sessile cells of Newman in single culture (first histogram), AA43 in single culture (second histogram), Newman and AA43 in ideal co-culture if the 2 species would not interfere each other (third histogram, percentages have been calculated considering the values of the first and second histograms), and Newman and AA43 in co-culture (fourth histogram). SA: S. aureus; PA: P. aeruginosa.

Figure 4 shows the percentage of planktonic and sessile cells of the two species in single and dual cultures. While Newman in pure culture presented the highest percentage of sessile cells, in dual culture was negatively affected by the presence of PA14 and AA2, and the biofilm composition of the co-culture reflected that of *P. aeruginosa* in pure culture (Figure 4A and 4B). A reciprocal influence was evident only for the pair represented by Newman and the late strain AA43 (Figure 4C). It is worth noting that in single species biofilm, the mucoid AA43 strain, even if apparently displaying a lower biofilm biomass compared to AA2 after staining with crystal violet, presented a higher percentage of sessile cells compared to AA2 (5.7% vs 3.4% respectively).

### Competition between *P. aeruginosa* and *S. aureus* in a mouse model of acute lung infection

**Figure 5:**
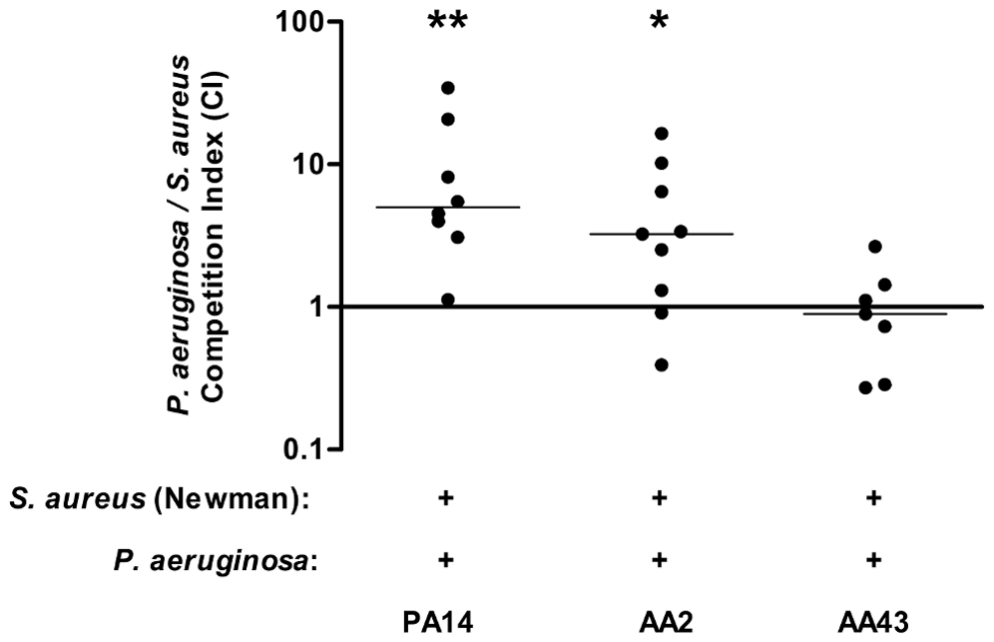
Competition between *P. aeruginosa* and *S. aureus* strains in a murine model of pneumoniae. Planktonic *S. aureus* strain Newman and *P. aeruginosa* clinical isolates AA2 and AA43 and reference strain PA14 were used to infect C57BL/6NCrlBR mice at a ratio of 1∶1. After 18 hours of acute infection lungs homogenates were plated on selective plates to determine *S. aureus* and *P. aeruginosa* CFU. Each circle represents the CI for a single animal in each group. A CI value equal to 1 indicates equal competition of the two species; a CI value significantly <1 indicates a competitive advantage of *S. aureus* that outcompetes *P. aeruginosa*; a CI value significantly >1 indicates a competitive advantage of *P. aeruginosa* that outcompetes *S. aureus*. Wilcoxon signed rank test of the null hypothesis that the distribution of CI is symmetric about 1 was performed. Statistically significant differences are indicated by symbols when present: *: p<0.05; **: p<0.01. The data are pooled from two or three independent experiments.

To test whether the observed differences in planktonic growth and biofilm formation *in vitro* would be relevant *in vivo*, a mouse model of acute pneumonia was used. Thus, we set up *in vivo* competition between *P. aeruginosa* and *S. aureus* in C57Bl/6NCrlBR mice challenged with 1×10^6^ CFU of *S. aureus* and *P. aeruginosa* strains mixed together at a 1∶1 ratio. Eighteen hours after infection, murine lungs were homogenized and plated. Differential CFU counting was performed to calculate the CI. Results show that *P. aeruginosa* reference strain PA14 and the early isolate AA2 outcompeted *S. aureus* strain Newman, as the CI, being significantly different from 1, indicated a competitive advantage of *P. aeruginosa* over *S. aureus* (PA14/Newman average CI = 5.0, p<0.01; AA2/Newman average CI = 3.3, p<0.05). Different results were obtained for the *P. aeruginosa* late isolate AA43 and Newman as the CI 18 hours after challenge was not significantly different from 1 (AA43/Newman CI = 0.9), indicating no competition in this case (Figure 5 and Table 2).

**Table 2:** Colonization of murine lungs with S. aureus and P. aeruginosa reference and clinical strains in competition experiments.

	PA14/Newman (n = 9^a^)	AA2/Newman (n = 9^a^)	AA43/Newman (n = 9^a^)
**Mortality, % (no. of dead/total mice)**	0 (0/9)	0 (0/9)	0 (0/9)
**Co-infected** b **, % (no. of co-infected/surviving mice)**	89 (8/9)	100 (9/9)	78 (7/9)
***P. aeruginosa*** ** infected** c **, % (no. of infected/surviving mice)**	100 (9/9)	100 (9/9)	78 (7/9)
***S. aureus*** ** infected** d **, % (no. of infected/surviving mice)**	89 (8/9)	100 (9/9)	78 (7/9)
**Total cfu/lung** e	3.3×10^4^	6.7×10^3^	5.8×10^3^
***P. aeruginosa*** ** cfu/lung** e	2.9×10^4^	4.5×10^3^	2.7×10^3^
***S. aureus*** ** cfu/lung** e	4.2×10^3^	2.2×10^3^	3.1×10^3^
**CI** f	5.0	3.3	0.9

a Pooled mice, analyzed in two independent experiments.

b Co-infected mice, surviving after 18 hours from challenge.

c Number of pooled mice infected with *P. aeruginosa* after 18 hours.

d Number of pooled mice infected with *S. aureus* after 18 hours.

e Median values are reported.

f Competition Index.

## Discussion

The goal of this study was to investigate the influence of *P. aeruginosa* adaptation to the CF lung on interaction with *S. aureus* in co-culture, during biofilm formation and mouse lung infection, in order to gain important insight on the interplay occurring between the two main pathogens of CF airways, which is still largely unknown. For this purpose, we used a panel of deeply genetically and phenotypically characterized *P. aeruginosa* clonal strains isolated from CF patients at different time points during CF chronic lung infection [33], [34].

We evaluated the inhibitory effect of eight *P. aeruginosa* lineages on *S. aureus*, including strains isolated both at early and late stage of chronic infection. A negative effect on *S. aureus* growth significantly associated with early-infecting *P. aeruginosa* strains was observed, while clonal late-infecting *P. aeruginosa* strains presented a significantly reduced or abolished virulence when co-cultivated with *S. aureus*. During chronic infection, *P. aeruginosa* undergoes adaptation to the CF lung, leading to patho-adaptive lineages that differ genotypically and phenotypically from the originally infecting strain. Such microevolution usually determines loss of motility, acquisition of mucoidy, antibiotic resistance and loss-of-function mutations in virulence genes, suggesting attenuation of virulence for CF adapted strains [30], [35], [36], [37]. Here we demonstrated for the first time that *P. aeruginosa* virulence traits affect also the interaction with other CF-related pathogen as *S. aureus*. As described for other traits, intra-clonal variation was observed both in clonal *P. aeruginosa* early strains and late strains isolated at the same time from the CF patients. One of the most striking characteristics of *P. aeruginosa* chronic lung infection in CF patients is the intense diversification of the bacterial population, leading to the co-existence of multiple phenotypes that may colonize different airways niches. Thus, the intra-clonal variation that we have observed is most likely the result of this process of genetic adaptation.

Under planktonic growth conditions, we have shown that both the reference *P. aeruginosa* strain PA14 and the clinical early strain AA2 strongly inhibited the growth of *S. aureus* during late logarithmic phase and stationary phase, without being influenced in their growth rate. Antagonism between microorganisms within a community could be attributed to simple competition for limited resources or to direct antagonistic effects [36]. There is evidence supporting antagonism between *P. aeruginosa* and *S. aureus*. Mashburn *et al.* demonstrated that *P. aeruginosa* can lyse *S. aureus* to use the iron released for its own growth [38]. Moreover, it has been reported that *S. aureus* is susceptible to an arsenal of respiratory inhibitors generated by *P. aeruginosa*, such as pyocyanin, hydrogen cyanide or alkyl-hydroxyquinoline N-oxides (HQNO), which are able to suppress the aerobic metabolism and growth of *S. aureus* [25], [39]. Interestingly, the late *P. aeruginosa* strain AA43, clonal to AA2, inhibited the growth of *S. aureus* at a lower extent, compared to AA2 and was not able to outcompete it. Besides being less virulent, AA43 was also negatively affected by the presence of *S. aureus* as its growth rate was significantly slowed down by *S. aureus* cells.

Despite the increasing interest on the crucial role of biofilm in CF infections, interspecies interactions of different organisms in mixed species biofilms are still poorly understood [27]. Here we have shown that in co-culture biofilms all *P. aeruginosa* strains were able to outcompete *S. aureus* in both sessile and planktonic fractions and the composition of the population in mixed biofilms was determined by *P. aeruginosa*, albeit to different extent. Also under biofilm growth conditions, the clonal late *P. aeruginosa* strains AA43 presented a different behavior in the presence of *S. aureus* compared to the early AA2 strain. In single species biofilm, the mucoid AA43 strain, even if apparently displaying a lower biofilm biomass compared to AA2 after staining with crystal violet, presented a higher percentage of sessile cells compared to AA2. This difference in biofilm production reflects the well documented phenotypic changes occurring in *P. aeruginosa* during the establishment of chronic infection. Indeed, *P. aeruginosa* strains isolated from CF patients at early stage of chronic infection are generally non-encapsulated and express a variety of virulence factors, whereas *P. aeruginosa* isolates from late stage typically lack virulence factors and convert to a mucoid phenotype, associated with greater biofilm formation and resistance to phagocytosis [37]. In apparent contradiction, also the early strain AA2 was able to produce biofilm. This could be explained considering the complexity of the microbial interactions in the CF lung, the presence of a diverse community of *P. aeruginosa* strains, and the many factors contributing to the formation of the biofilm matrix of *P. aeruginosa*, besides alginate production. In addition not all adapted isolates are mucoid and also early not adapted strains could produce biofilm exploiting other biofilm matrix molecules [40]. In agreement with data obtained in planktonic co-cultures, AA2 strongly inhibited the growth of *S. aureus* in mixed biofilm, without being affected. Qazi *et al.* demonstrated that factors related to biofilm formation are down-regulated in *S. aureus* in response to *P. aeruginosa* presence, consistently with our results [41]. Compared to AA2, *P. aeruginosa* AA43 inhibited *S. aureus* growth at lower extent, determining a reduction of *S. aureus* CFU count of about 1 and 2 log in planktonic and sessile fractions respectively, when measured against *S. aureus* individual biofilm. Moreover, the capacity to produce biofilm of AA43 was negatively affected by the presence of *S. aureus*, confirming its attenuated virulence and susceptibility to competitor organism.

Although several studies using *in vitro* models demonstrated an inhibitory effect of *P. aeruginosa* on the growth of also highly virulent *S. aureus* strains such as USA 300, in line with our results [27], [42], [43], *in vivo* models show contradictory results [42], [44]. We further investigated *S. aureus*/*P. aeruginosa* reciprocal interaction setting up a murine model of acute lung co-infection. In agreement with *in vitro* data, the reference strain PA14 and the early CF clinical isolate AA2, after 18 hours of co-infection, inhibited *S. aureus*, while the late CF clinical isolate AA43 did not outcompete *S. aureus*.

It is known that environmental and early clinical isolates of *P. aeruginosa* are equipped with a repertoire of virulence factors and, among them, also substances with anti-bacterial activity, these factors are selected against during the adaptation process to the CF airways environment. The results obtained in the acute pneumonia model, in which an early isolate is able to inhibit the growth of another pathogen, while its clonal adapted strain is no longer able to do so, strengthen the loss of anti-bacterial factors during adaptation.

Our data underline the importance of bacterial interactions in lung infection and in particular of the complexity of the interactions of different pathogens that coexist in the CF airways. However, given the genetic adaptation process of *P. aeruginosa* that leads to the selection of different patho-adaptive variants, descending from the initial infecting clone, further combinations of clonal lineages of early and late isolates should be tested to strengthen our *in vivo* data. Moreover, considering that the adaptation process during chronic infection involves also *S. aureus*, other experiments using clinical early and late *S. aureus* strains as well as adapted phenotypes such as small colony variants should be performed. Our results showing the influence of adaptation on the reciprocal interactions between *S. aureus* and *P. aeruginosa* deserve further investigations including the host response and the effect of environmental conditions, such as microaerobic and anaerobic conditions, on pathogens interactions, using both *in vitro* and *in vivo* models of chronic infection that better mirror the progression of CF lung disease.

## Materials and Methods

### Animals and ethics statement

Animal studies were conducted according to protocols approved by the San Raffaele Scientific Institute (Milan, Italy) Institutional Animal Care and Use Committee (IACUC, Number 444) and adhered strictly to the Italian Ministry of Health guidelines for the use and care of experimental animals. All efforts were made to minimize the number of animals used and their suffering.

Research on *P. aeruginosa* bacterial isolates from the individuals with CF has been approved by the responsible physician at the CF center at Hannover Medical School, Germany. All patients gave informed consent before the sample collection. Approval for storing of biological materials was obtained by the Hannover Medical School, Germany.

### Bacterial strains

For *S. aureus*, Newman and SH1000 reference strains, were used in the study [45], [46]. Two *P. aeruginosa* reference strains, PA14 [47] and PAO1 [48], and 8 clonal lineages of *P. aeruginosa* clinical strains from CF patients (AA, SG, NN, BT, TR, MF, KK, BST), including strains isolated at the onset of chronic colonization (early: AA2, SG1, NN2, BT1, BT2, TR1, TR2, MF1, MF2, KK1, KK2, BST2) or several years after acquisition and before patient's death (late: AA43, SG57, SG58, NN83, BT72, BT73, TR66, TR67, MF51, KK71, KK72, BST44) were used in this study [23]. Clonality of strains, assessed by Pulsed Field Gel Electrophoresis and multiple phenotypic traits, including motility, mucoid phenotype, *LasR* phenotype, and pyocyanin secretion, have been determined and previously reported [23], [32].

### *S. aureus* growth inhibition on agar surface

*S. aureus* cultures (Newman and SH1000) grown overnight in Luria-Bertani broth (LB, Difco™) were normalized to 0.5 OD_600_ and uniformly spread on Mueller-Hinton agar plate (MH, Difco™) by using a cotton swab. Then 5 µl of *P. aeruginosa* culture, grown overnight in LB broth and normalized to 0.5 OD_600_, were added to the *S. aureus* lawn followed by incubation overnight at 37°C [25]. The same procedure was performed spotting *S. aureus* culture on *P. aeruginosa* lawn. The following *P. aeruginosa* clonal lineages, including early and late clinical strains, were tested: AA, SG, NN, BT, TR, MF, KK and BST (for details see paragraph “Bacterial strains”). As *P. aeruginosa* reference strains we used PA14 and PAO1. The inhibition score was defined as follows: “no inhibition” when no halo was observed around the spot of *P. aeruginosa* that measures 9 mm; “weak inhibition” indicated an inhibition halo ≤15 mm; “strong inhibition” indicated an inhibition halo >15 mm and ≤25 mm; “very strong inhibition” indicated an inhibition halo >25 mm. The choice for inhibition strength ranges was based on preliminary assays performed using the lawn of about 30 *S. aureus* strains (including both reference and clinical strains of different origin) and spotting about 60 *P. aeruginosa* strains (both reference and clinical strains) on the different lawns.

### Planktonic mono-culture and co-culture growth curves

All growth curves were performed in 30 ml of nutrient-rich not selective medium, Brain-Heart Infusion broth (BHI, Difco™), at 37°C with shaking (180 rpm). The following strains were tested: *S. aureus* (Newman, SH1000), *P. aeruginosa* (PA14, AA2 and AA43). Strains were grown overnight in BHI and subcultured in fresh medium for 2.5 hours to reach the mid-exponential phase of growth. Bacteria were centrifuged, pellet was resuspended in fresh medium and the OD_600_ was measured to adjust the concentration of bacteria. For co-cultures each pair of *S. aureus* and *P. aeruginosa* strains were inoculated at equal ratio (1 OD_600_, optical density) from mid-exponential phase pure cultures and incubated at 37°C for 24 hours. Pure cultures of each organism were used for comparative purposes. At different time points (0, 0.5, 1, 2, 3, 4, 6, 8, 12 and 24 hours), samples were taken, serially diluted in sterile phosphate-buffered saline (PBS) and plated onto Mannitol Salt agar (MSA, Difco™) and Pseudomonas Isolation agar (PIA, Difco™) to discriminate the two bacterial species. The agar plates were incubated for 24 hours at 37°C and colony forming units (CFU) were enumerated. Each experiment was repeated three times independently. The competition index (CI) for mixed culture was calculated as *P. aeruginosa*-to-*S. aureus* ratio within the output sample, divided by the corresponding ratio for the input (inoculum at time t = 0), as described by Macho and colleagues [49]. To allow an easier comparison between the variations observed in single versus mixed cultures a CI-like index, the Relative Increase Ratio (RIR) was calculated as *P. aeruginosa*-to-*S. aureus* ratio within the output sample, divided by the corresponding ratio in the inoculum, using growth results from pure cultures [49]. As the RIR is calculated on the results obtained from single growth curves, only a CI that differs statistically from the RIR of the same time-point can be considered a result of a significant competition between the species [49].

### Biofilm production

Biofilm production in static conditions was visualized by crystal violet (CV) staining as previously described [34]. The following *S. aureus* and *P. aeruginosa* strains were tested: Newman, PA14, AA2 and AA43. Strains were grown overnight in Nutrient Broth (NB, Difco™) and subcultured in fresh medium for 2.5 hours to reach the mid-exponential phase of growth. Bacteria were centrifuged, pellet was washed with PBS, resuspended in fresh medium and the OD_600_ was measured to adjust the concentration of bacteria [34]. Experiments were performed in triplicate and repeated three times independently. The data were then averaged and the standard deviation was calculated.

To correlate the growth in the planktonic fraction with biofilm formation, the planktonic cell fractions, which were transferred to new microtiter plates, were quantified by plating serial dilutions on MSA and PIA agar plates. To enumerate the sessile cells of *S. aureus* and *P. aeruginosa*, the wells were rinsed three times with 200 µl of PBS to remove non-adherent and weakly adherent bacteria. Then, the biofilm was removed by scraping the surface of each well with 1 ml PBS and the recovered cells were suspended by vortexing for 30 sec. The number of sessile cells was determined by plating serial dilutions on MSA and PIA agar plates. To ensure the complete detachment of the bacteria, CV (1%) assay was performed on each of the wells scraped, and absorbance determined at 595 nm.

### Mouse model of acute lung single and co-infection

Experiment of acute infection with *S. aureus* and *P. aeruginosa* strains were performed using C57Bl/6NCrlBR male mice (20–22 gr), purchased by Charles River, with minor modification to previous published protocols [20], [30]. For the co-infections, *P. aeruginosa* referent strain PA14 and clinical isolates AA2 and AA43, and *S. aureus* referent strain Newman, grown at middle exponential phase, were recovered by centrifugation and resuspended in PBS to the desired sub-lethal dose for infection of 1×10^6^ CFU both for *P. aeruginosa* and *S. aureus* and mixed together at a ratio of 1∶1.

C57Bl/6NCrlBR mice were anesthetized by an intraperitoneal injection of a solution of 2.5% Avertin (2,2,2-tribromethanol, 97%; Sigma Aldrich) in 0.9% NaCl and administered in a volume of 0.015 mlg^−1^ body weight. Trachea was directly visualized by a ventral midline incision, exposed and intubated with a sterile, flexible 22-g cannula (Becton, Dickinson, Italy) attached to a 1 ml syringe. Co-infection was established with a 60 µl inoculum implanted via the cannula into the lung, with both lobes inoculated. Mice were also infected with 1×10^6^ CFU of planktonic *P. aeruginosa* or *S. aureus* for comparative purposes.

After 18 hours from infection, mice were euthanized and murine lungs were aseptically excised, homogenized and plated onto MSA and PIA plates for differential CFU counting. The competition index (CI) was calculated as the ratio of *P. aeruginosa* to *S. aureus* bacteria recovered from the murine lungs after 18 hours from infection adjusted by the input ratio that was inoculated in each animal (*in vivo* CI). A CI value equal to 1 indicates equal competition of the two species; a CI value significantly <1 indicates a competitive advantage of *S. aureus* that outcompetes *P. aeruginosa*; a CI value significantly >1 indicates a competitive advantage of *P. aeruginosa* that outcompetes *S. aureus*.

### Statistical analysis

*In vitro* agar growth inhibition data were analyzed by means of a LME (Linear Mixed effect model) separated for Newman and SH1000. Response variable was inhibition and covariates were groups (early versus late) and a random effect on patient to account for lineages heterogeneity. To analyze batch co-culture data reported in Figure 1A, 1C, 1E the CFU/ml values were transformed using a log10 function. Data retrieved from single and co-culture experiments showed a similar starting point (estimated by intercept parameter A) and different behavior in some settings over time , leading to different plateau values (estimated by parameter B) at the end of the follow-up period. This suggested to use a nonlinear mixed-effect model (ref), (with the non-linearity described by a four-parameters logistic regression function) to estimate the log10(CFU/ml) trend, modelled as it follows: A+B/{1+exp[(C−x)/exp(D)]}.

This kind of model is widely used for growth curve modeling. Since the parameter A represents horizontal asymptote relative to the starting point, we assign a random effect (representing the heterogeneity among experiments) on this parameter to include heterogeneity among experiments. Parameter B represents the horizontal asymptote relative to the final plateau; we studied the possible influence of single/co-culture (described by its indicator variable), in order to test the hypothesis of different plateau at the end of the follow up. This represents the main effect of interest and its significance was tested comparing likelihood with and without it. Parameter C is the inflection point and has been estimated using a maximum likelihood principle. Parameter D is strictly connected to the so called scale parameter and represents the growth rate of the logistic function. A fixed effect common for single single and co-culture was estimated. RIR and CI indexes were analyzed using Student's t-test and the null hypothesis: mean CI was not significantly different from mean RIR [49]. *In vitro* biofilm data (OD values) were analyzed using Two-tailed Student's t-test [34]. CFU biofilm data were analyzed by means of Mann–Whitney test, a non-parametric procedure to evaluate a null hypothesis that two populations are the same against an alternative that one population tends to have larger values than the other. Competition index (CI) of *in vivo* experiments was calculated adapting the methods previously published [20]. To assess bacterial competition Wilcoxon signed rank test of the null hypothesis that the distribution of CI is symmetric about 1 was used. Significance was set at the usual level 0.05. All statistical analyses were performed using R 2.15.2 (http://www.R-project.org/).

## Supporting Information

Table S1 ***In vitro*** **growth inhibition of** ***P. aeruginosa*** **.**
(DOCX)

Figure S1 **Single and dual species batch growth curves and competition index values.** S. aureus strain (SH1000) and P. aeruginosa strains (PA14 and two clinical early and late isolates from a CF patient AA2 and AA43) were grown for 24 hours in BHI in single culture and in co-culture after inoculation at equal ratio from mid-exponential phase pure cultures. Growth rate was monitored by colony count after plating on selective media for both species. Results are represented as the mean of values obtained from three independent experiments. The error bars indicate the standard deviations. A nonlinear mixed-effect model was fitted, using a four-parameters logistic regression function. Panel A: growth curves of SH1000 in pure culture and in co-culture with PA14; Panel B: Competition index (CI) and Relative Increase Ratio (RIR) calculated from single and dual cultures of SH1000 and PA14; Panel C: growth curves of SH1000 in pure culture and in co-culture with AA2; Panel D: CI and RIR calculated from single and dual cultures of SH1000 and AA2; Panel E: growth curves of SH1000 in pure culture and in co-culture with AA43; Panel F: CI and RIR calculated from single and dual cultures of SH1000 and AA43. Each value represents the mean of CI and RIR values from three independent experiments and the bars indicate standard deviation. Statistically significant differences in Student's t test and in nonlinear mixed-effect model are indicated by symbols when present: *: p<0.05; ***: p<0.001.
(TIF)
