# Supplementary material for: Adaptation of Pseudomonas aeruginosa in Cystic Fibrosis Airways Influences Virulence of Staphylococcus aureus In Vitro and Murine Models of Co-Infection
Source: PLoS One. 2014 Mar 6;9(3):e89614. doi: 10.1371/journal.pone.0089614 (PMC3945726; doi:10.1371/journal.pone.0089614)
Supplement: Table S1 — In vitro growth inhibition of P. aeruginosa . (DOCX) [file pone.0089614.s001.docx]

**SUPPLEMENTARY MATERIALS**

**Table S1. *In vitro* growth inhibition of *P. aeruginosa*.**

Twenty-four *P. aeruginosa* isolates were collected from eight individuals with CF (SG, NN, BT, AA, TR, MF, KK, BST) at the onset of chronic colonization (numbered 1-2) or after 4.5-16.3 years of colonization (numbered 43-83). PAO1 and PA14 were included as reference strains. 5 µl spots of *S. aureus* (Newman and SH1000) overnight cultures, normalized to 0.5 OD, were added to *P. aeruginosa* lawn (normalized to 0.5 OD) on Mueller-Hinton agar and incubated overnight at 37°C. The table summarizes the results obtained: “no” indicates absence of inhibition halo (9 mm is the diameter of the *S. aureus* spot). * Indicates mucoid phenotype. ^#^ Indicates hypermutable phenotype. For statistical analysis see “Results”.

| ***P. aeruginosa* lawn** | ***S. aureus* spot (Newman)**  **(inhibition halo)** | ***S. aureus* spot (SH1000)**  **(inhibition halo)** |
| --- | --- | --- |
| PAO1 | no (9 mm) | no (9 mm) |
| PA14 | no (9 mm) | no (9 mm) |
| SG1 | no (9 mm) | no (9 mm) |
| SG57* | no (9 mm) | no (9 mm) |
| SG58* | no (9 mm) | no (9 mm) |
| NN2 | no (9 mm) | no (9 mm) |
| NN83^#^* | no (9 mm) | no (9 mm) |
| BT1^#^ | no (9 mm) | no (9 mm) |
| BT2 | no (9 mm) | no (9 mm) |
| BT72* | no (9 mm) | no (9 mm) |
| BT73* | no (9 mm) | no (9 mm) |
| AA2 | no (9 mm) | no (9 mm) |
| AA43* | no (9 mm) | no (9 mm) |
| TR1 | no (9 mm) | no (9 mm) |
| TR2 | no (9 mm) | no (9 mm) |
| TR66* | no (9 mm) | no (9 mm) |
| TR67* | no (9 mm) | no (9 mm) |
| MF1 | no (9 mm) | no (9 mm) |
| MF2^#^ | no (9 mm) | no (9 mm) |
| MF51* | no (9 mm) | no (9 mm) |
| KK1 | no (9 mm) | no (9 mm) |
| KK2 | no (9 mm) | no (9 mm) |
| KK71* | no (9 mm) | no (9 mm) |
| KK72* | no (9 mm) | no (9 mm) |
| BST2 | no (9 mm) | no (9 mm) |
| BST44^#^* | no (9 mm) | no (9 mm) |
