# Supplementary figures and images for: Adaptation of Pseudomonas aeruginosa in Cystic Fibrosis Airways Influences Virulence of Staphylococcus aureus In Vitro and Murine Models of Co-Infection
Source: PLoS One. 2014 Mar 6;9(3):e89614. doi: 10.1371/journal.pone.0089614 (PMC3945726; doi:10.1371/journal.pone.0089614)

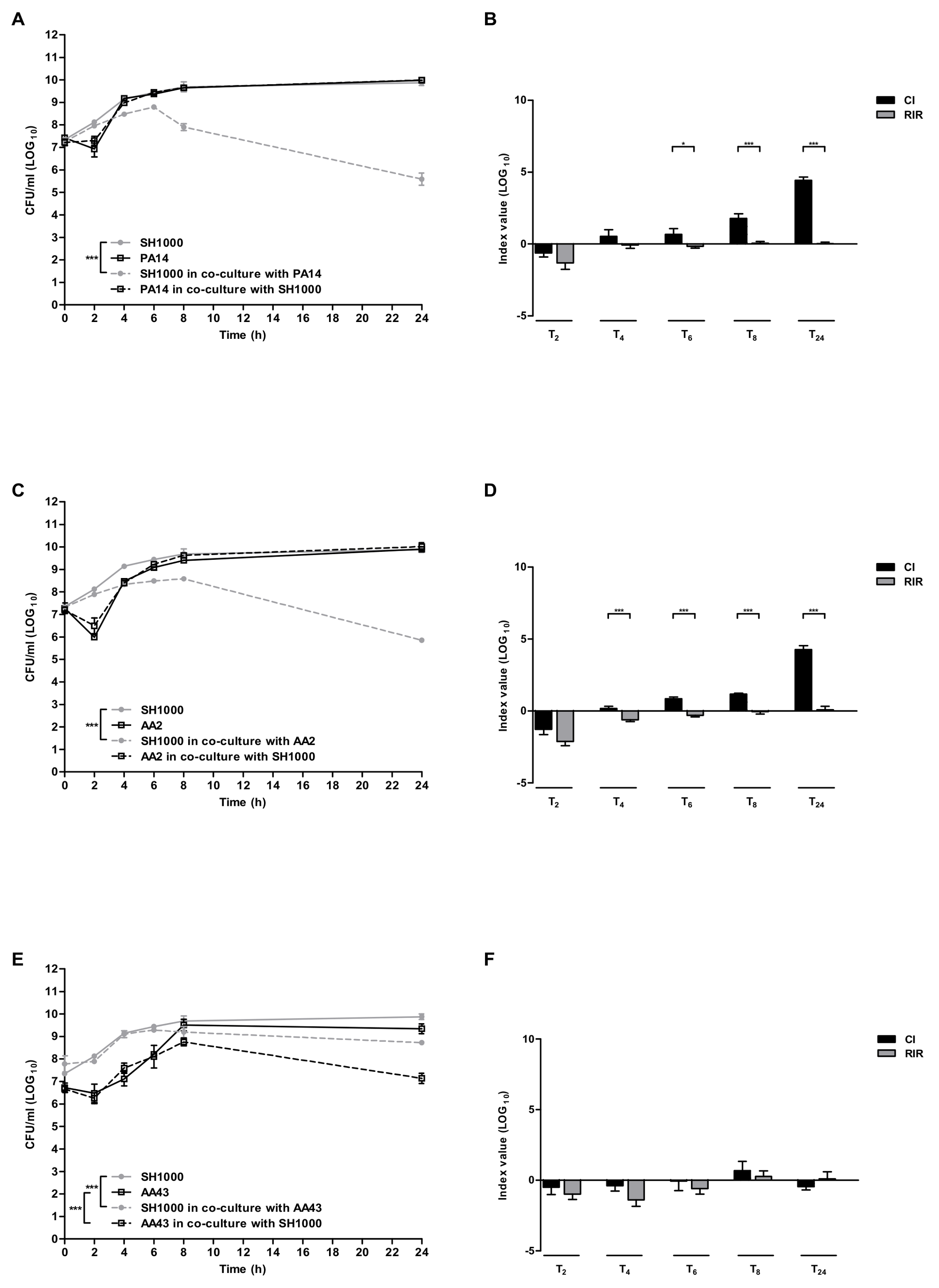

Supplement: Figure S1 — Single and dual species batch growth curves and competition index values. S. aureus strain (SH1000) and P. aeruginosa strains (PA14 and two clinical early and late isolates from a CF patient AA2 and AA43) were grown for 24 hours in BHI in single culture and in co-culture after inoculation at equal ratio from mid-exponential phase pure cultures. Growth rate was monitored by colony count after plating on selective media for both species. Results are represented as the mean of values obtained from three independent experiments. The error bars indicate the standard deviations. A nonlinear mixed-effect model was fitted, using a four-parameters logistic regression function. Panel A: growth curves of SH1000 in pure culture and in co-culture with PA14; Panel B: Competition index (CI) and Relative Increase Ratio (RIR) calculated from single and dual cultures of SH1000 and PA14; Panel C: growth curves of SH1000 in pure culture and in co-culture with AA2; Panel D: CI and RIR calculated from single and dual cultures of SH1000 and AA2; Panel E: growth curves of SH1000 in pure culture and in co-culture with AA43; Panel F: CI and RIR calculated from single and dual cultures of SH1000 and AA43. Each value represents the mean of CI and RIR values from three independent experiments and the bars indicate standard deviation. Statistically significant differences in Student's t test and in nonlinear mixed-effect model are indicated by symbols when present: *: p<0.05; ***: p<0.001. (TIF) [file pone.0089614.s002.tif]
